# HTLV-1 Infection and Pathogenesis: New Insights from Cellular and Animal Models

**DOI:** 10.3390/ijms22158001

**Published:** 2021-07-27

**Authors:** Greta Forlani, Mariam Shallak, Roberto Sergio Accolla, Maria Grazia Romanelli

**Affiliations:** 1 Laboratory of General Pathology and Immunology “Giovanna Tosi”, Department of Medicine and Surgery, University of Insubria, 21100 Varese, Italy; greta.forlani@uninsubria.it (G.F.); mshallak@uninsubria.it (M.S.); roberto.accolla@uninsubria.it (R.S.A.); 2 Department of Biosciences, Biomedicine and Movement Sciences, University of Verona, 37134 Verona, Italy

**Keywords:** HTLV, humanized mice, ATL, HAM/TSP, Tax, HBZ, BLV, STLV, CRISPR, NF-κB, CIITA, restriction factors

## Abstract

Since the discovery of the human T-cell leukemia virus-1 (HTLV-1), cellular and animal models have provided invaluable contributions in the knowledge of viral infection, transmission and progression of HTLV-associated diseases. HTLV-1 is the causative agent of the aggressive adult T-cell leukemia/lymphoma and inflammatory diseases such as the HTLV-1 associated myelopathy/tropical spastic paraparesis (HAM/TSP). Cell models contribute to defining the role of HTLV proteins, as well as the mechanisms of cell-to-cell transmission of the virus. Otherwise, selected and engineered animal models are currently applied to recapitulate in vivo the HTLV-1 associated pathogenesis and to verify the effectiveness of viral therapy and host immune response. Here we review the current cell models for studying virus–host interaction, cellular restriction factors and cell pathway deregulation mediated by HTLV products. We recapitulate the most effective animal models applied to investigate the pathogenesis of HTLV-1-associated diseases such as transgenic and humanized mice, rabbit and monkey models. Finally, we summarize the studies on STLV and BLV, two closely related HTLV-1 viruses in animals. The most recent anticancer and HAM/TSP therapies are also discussed in view of the most reliable experimental models that may accelerate the translation from the experimental findings to effective therapies in infected patients.

## 1. Introduction

Human T-cell leukemia virus type 1 (HTLV-1), isolated in the early 1980s from T cell lines derived from patients with cutaneous T-cell lymphoma and human adult T-cell leukemia, was the first human retrovirus to be discovered [1,2]. Since then, four HTLV types have been isolated in humans and have been phylogenetically associated with the simian STLV viruses [3,4]. In contrast to the HIV retrovirus, no efficient therapy is yet available to avoid the onset of the most alarming diseases caused by HTLV-1. In vivo, HTLV-1 infects mainly CD4+ T cells, the key cells in the triggering and establishment of the adaptive immune response. Besides being the etiological agent of adult T-cell leukemia/lymphoma (ATL), in 3–5% of infected subjects, HTLV-1 causes immune hypersensitivity conditions like arthritis, uveitis, and most importantly, the HTLV-1-associated myelopathy/tropical spastic paraparesis (HAM/TSP), a fatal chronic inflammatory neurological disorder [5,6,7,8]. Most infected people, however, remain asymptomatic, highlighting the role of the immune system in the control of infection [9,10,11]. Worldwide, more than 20 million subjects are infected by HTLV and, despite advances in treatment, patients with aggressive ATL generally have a poor prognosis [12,13].

**Figure 1:**
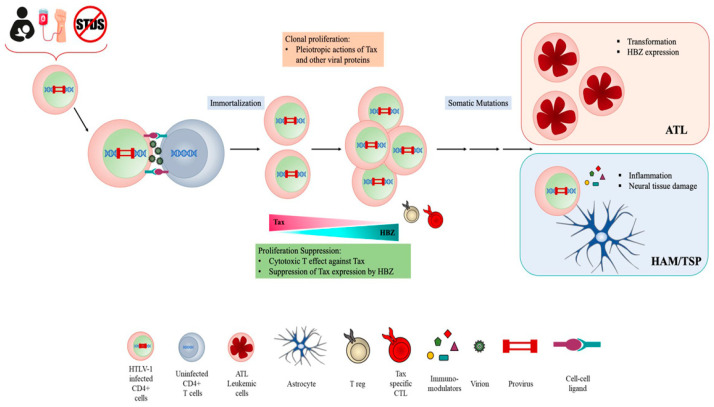
HTLV cell infection and transmission.

HTLV-1 persistent infection is likely associated with the ability of the virus to evade the host’s immune response. Immune evasion might correlate with high proviral load and thus to disease outcome. HTLV-1 infection occurs exclusively through cell-to-cell contact, and the number of infected cells in vivo significantly impacts on viral spreading [14]. After primary infection, the clonal expansion of infected cells, rather than the novo infection of cells, represents the main route for HTLV-1 to establish persistent and chronic infection [15] (Figure 1). 

The proviral genome is integrated into the host genome and contains, in addition to structural proteins Gag, Pol and Env, a unique pX region coding for several regulatory and nonstructural proteins Tax, Rex, p12, p13, p30 and HTLV-1 basic zipper protein (HBZ), which is encoded by the antisense viral transcript. Among them, Tax and HBZ are thought to play key roles in HTLV-1 infection and oncogenesis. Transgenic mice expressing Tax or HBZ develop neoplastic diseases, indicating that they function as oncogenes [16,17,18,19]. Tax is a potent activator of viral transcription and exerts pleiotropic effects on cell signaling deregulating different cellular pathways thus mainly contributing to HTLV-1 induced neoplastic transformation. However, the frequent loss of Tax expression from ATL cells suggests that the viral protein is mainly involved in the onset of leukemic transformation. By contrast, HBZ is ubiquitously expressed, playing a crucial role in the maintenance of oncogenic process and disease progression. Furthermore, recent reports from our laboratory have demonstrated that HBZ subcellular localization could be a prognostic marker of HTLV-1-disease progression, as HBZ is expressed solely in the cytoplasm of asymptomatic carriers (AC) and HAM/TSP subjects, while in tumor cells isolated from leukemic patients, it is also present in the nucleus [20,21,22]. HBZ antagonizes many of the activities of Tax and suppresses Tax-induced viral transcription, thus the interaction between Tax and HBZ may significantly affect the outcome of HTLV-1 infection [23,24,25].

Although intensive studies in recent years have contributed to shedding light on the mechanisms of viral replication and host response, several aspects of HTLV-1 pathogenesis remain poorly understood, including the intimate molecular mechanism(s) of tumorigenesis, the progression of HTLV-1 leukemia toward the aggressive form, and the possibility of enhancing the host response to avoid or at least delay disease onset.

Here we will discuss the complex landscape of host–viral interactions in the context of HTLV-1 infection, pointing out key experimental systems (e.g., cells and animal models) suitable for the study of HTLV-1 viral transmission, disease pathogenesis, and treatment and their recent contribution to the advancement of knowledge of HTLV infection.

## 2. Cell Models

Initially, most of the data related to HTLV-1 infection, mode of transmission, pathogenesis as well as the description of the cellular pathways targeted by viral proteins were derived from studies of viral factors over-expressed in cells that are not targets of infection or from viral particles produced in cells transduced with the HTLV-1 proviral genome. Subsequently, the ability of HTLV-1 to infect different types of cells in vitro and transmit the infection via cell-to-cell contact has made it possible to generate HTLV-1-producing cell lines (such as MT-2, MT-4, C91-PL and SP) by coculturing leukemic cells from ATL patients with human cord blood lymphocytes isolated from healthy subjects. HTLV-1-infected T cell lines have also been established from ATL patients, such as ATL-2 cells, MT-1, ATL-T, TLOm1, ED, ATL35-T, and ATL-55T. Commonly used cell lines in HTLV studies are listed in Table 1 [11,26,27,28,29,30,31].

Studies conducted in primary T lymphocytes isolated from the peripheral blood of HTLV-1-infected individuals have added important elements to the knowledge of the mechanism of HTLV-1 pathogenesis, as these cells showed increased spontaneous IL-2-dependent proliferation when cultured in vitro. However, over time, proliferation often becomes independent of (IL-2), and these cell cultures generally represent outgrowths of clones that do not predominate in the leukemic patients, but rather are selected for growth in culture. Moreover, primary ATL cells express CD3, CD4 and CD25 but not CD7 in most cases, and 10–15% express both CD4 and CD8. Generally, signal transduction and gene expression alterations observed in ATL cells were also found in HTLV-1-infected and virus-expressing T cells, although viral genes, except for antisense transcripts, such as HBZ, are not expressed in ATL cells. Another important limitation in the study of HTLV-1 is the absence of a reliable system to measure de novo infection, due to the fact that HTLV-1 cell-free virus preparations are largely not infectious [31]. Here we will review the most recent contribution derived by studies based on cellular models on HTLV-1 infection and pathogenesis. 

### 2.1. HTLV-1 Infection and Cell-to-Cell Transmission

As mentioned above, HTLV-1 transmission occurs through contact with infected cells present in body fluids such as in maternal milk, semen, or blood.

**Figure 2:**
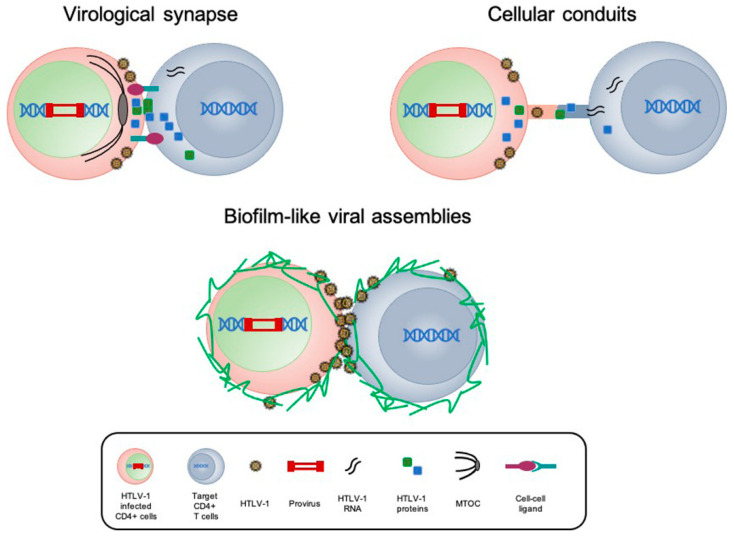
Models of HTLV-1 cell-to-cell transmission.

In vitro studies conducted by coculturing a target cell with HTLV-1-infected T cell line confirmed that cell-free virus is poorly infectious and a virus-induced specialized cell-to-cell contact, based on specific interactions between cellular and viral proteins, is needed for an efficient virus transmission [32]. Two types of cell–cell contacts seem to be critical for HTLV-1 transmission: long cellular connections, including cellular conduits or tunneling nanotubes (TNTs) and virological synapse [33] (Figure 2).

The first mode of HTLV-1 transmission in cell models was reported by Iagakura et al. (2003), who observed the formation of virological synapse (VS) between fresh peripheral blood mononuclear cells (PBMC) isolated from HTLV-1-infected patients. Confocal microscopy studies have revealed that HTLV-1 Gag, Env proteins and genomic viral RNAs were unpolarized in isolated T cells but accumulated at cell–cell junction and transferred to uninfected T cells within 2 hours after contact. The formation of VS and the transport of viral proteins towards the VS and into the target cell requires the integrity of the microtubule cytoskeleton. The polarization of the microtubule organizing centers (MTOC) of the infected cell towards the cell–cell contact may be promoted by both the viral transactivator Tax-1 and the cell adhesion molecule ICAM-1, which is also induced by Tax-1 [14]. Consistent with these observations are results suggesting that besides Tax, HBZ also promotes HTLV-1 infectivity by upregulating ICAM-1, thus contributing to homotypic aggregation of HBZ-expressing Jurkat clones. Moreover, by using luciferase-based infection assays with HTLV-1-infected SLB1 cells deleted for HBZ, significantly lower luciferase activity was observed from cocultures containing HBZ knock-down cells than from control cells, supporting the role of HBZ in facilitating HTLV-1 infection [34]. 

Electron tomography studies on CD4+ T cells isolated from HTLV-1-infected individuals and MS-9 chronically HTLV-1-infected cell line have revealed the ultrastructure of the HTLV-1 VS and the requirement for cell contact and the HTLV-1-Env protein for HTLV-1 [14,35,36]. Viral transmission at VS could also occur through the generation of a biofilm-like structure in the extracellular matrix (ECM), called viral biofilm (VB) which favors the transfer of viruses accumulated at the surface of infected cell. HTLV-1 transmission via VBs represents a major route of transmission in vitro, since removal of biofilms severely impairs cell-to-cell transmission [37]. It has been suggested that Tax-1 might contribute to the formation of VB by inducing the expression of the actin-binding protein Fascin, which plays a significant role in enhancing HTLV-1 gag protein transfer to uninfected target cells. Furthermore, confocal microscopy studies conducted in HTLV-1-infected MS-9 cells cocultured with Jurkat T cells revealed that Fascin colocalized with gag in long-distance connections between chronically infected and newly infected T cells, suggesting that Fascin could be important for the transport of viral proteins to foster polarized budding, virus release and cell-to-cell transmission of HTLV-1 [38]. The role of Tax-1 HTLV-1 transmission by VB formation was also sustained by the observation that Tax-1 increases the expression of Collagen IV (COL4), a crucial component of VB, in either HTLV-1-infected (MT-2, C91-PL and HUT-102) or Tax-transformed T cell lines (Tesi, Tri and TAXI-1). Consistently, imaging and flow cytometry studies have revealed that CRISPR/Cas9 knockout of COL4 in the chronically HTLV-1-infected T cell line C91PL significantly impacts HTLV-1 Gag-p19 transfer to target cells, indicating an important role of COL4 in HTLV-1 cell-to-cell transmission [30].

Recent studies by coculturing HCT-5 cells with salivary gland epithelial cells have suggested that VB might facilitate the initial phase of transmission of HTLV-1 virions to non-immune cells, such as salivary gland epithelial cells [39,40]. 

Cellular conduits induced in the infected cells by the accessory protein p8 represent another route for HTLV-1 transmission. In addition to its role in increasing adhesion molecules association, such as Lymphocyte Function Associated Antigen 1/Intracellular Adhesion Molecules 1 (LFA- 1/ICAM-1) interactions, the combined use of live imaging and electron transmission microscopy (TEM) demonstrated that overexpression of p8 in MT-2 chronically infected cells increases the number and length of these conduits, as well as the number of contacts between infected and uninfected cells [41]. A recent report aimed at identifying cellular interaction partners of p8, responsible for its rapid transfer through cellular conduits, demonstrated that Vasodilator-stimulated phosphoprotein (VASP) interacts with p8 and this interaction is crucial for p8 transfer between cells [42]. Imaging and flow cytometry studies have revealed that silencing of both endogenous and overexpressed VASP by RNA interference or by CRISPR/Cas9 reduced p8 transfer to the cell surface and to target Jurkat T cells. In addition, stable repression of VASP by RNA interference in chronically infected MT-2 cells impaired not only p8, but most importantly HTLV-1 Gag transfer to target Jurkat T cells, suggesting that HTLV-1 cell-to-cell transmission depends on VASP containing cellular conduits [38,43,44]. The viral protein p8 has also been shown to contribute to HTLV-1 transmission through the formation of actin, but not tubulin, containing structures, defined as tunneling nanotubes (TNTs). Immunofluorescence and confocal microscopy studies have indicated that MT-2 formed TNT with noninfected T cells or monocytes containing gag, Tax-1 and p8, and the number of TNT was significantly reduced in cells treated with cytarabine, an inhibitor of TNT formation [33]. 

In vivo HTLV-1 preferentially infects CD4+ T cells co-expressing CCR4 receptor and induces functional changes in the infected cells, mainly driven by oncoprotein Tax-1 [7]. Besides CD4+ T cells and, to a lesser extent, CD8+ T lymphocytes, other immune cell types such as myeloid dendritic cells (DC) and monocyte-derived dendritic cells (MDDC) have been shown to be productively infected by HTLV-1 in vitro and release viral particles in culture supernatants. Interestingly, infected DC could efficiently transmit the infection to T cells through cell–cell contact or VB accumulated at the surface of infected donor cells, thus contributing to viral dissemination and, concomitantly, being inhibited in their antigen-presenting function [45]. As a result, HTLV-1-infected DC could not prime naïve T cells, thus preventing their final effector function [46].

In fact, monocytes obtained from ATL patients differentiate poorly into monocyte-derived dendritic cells (MDDCs) in vitro, have a reduced ability to present antigen, and have altered capacities to stimulate proliferation of allogeneic T lymphocytes [47].

### 2.2. HTLV-1 Dissemination

In vivo, HTLV-1 spreads through two different mechanisms: neo-infection of target cells or clonal proliferation of Tax-1-immortalized cells [7]. The activation of Tax-1 specific CTL and the direct inactivation of viral RNA contribute to the establishment of chronic infection, thus inhibiting viral replication. Recent reports have shown that extracellular vesicles (EV) isolated from HTLV-1-infected cell lines contain Tax-1 and can also be isolated from HAM/TSP patient PBMCs and cerebrospinal fluid (CSF) samples [48]. Moreover, by using ionizing radiation to activate the virus in HTLV-1-infected HUT-102 cells it has been shown that EV release is increased. Fluorescent microscopy studies have shown that EV derived from HTLV-1-infected cells, such as MT-2, MT-4 and HUT-102, were found to colocalize with cell membranes of co-cultured uninfected cells, thus suggesting a possible mechanism of trans-cell Tax-1-dependent activation without infection. Furthermore, by using neutralizing antibody, it was shown that both CD45 and ICAM-1 could be considered a crucial molecular target in EV-mediated cell-to-cell contact, since their inhibition potentially suppresses viral transmission in PBMCs [49].

### 2.3. Restriction Factors

The interaction between HTLV-1 and the host immune response plays an important role in the outcome of HTLV-1-induced diseases [50]. The first line of defense against viral infection is represented by restriction factors (RF), host anti-viral components of intrinsic immunity that block viral replication and spreading. These cellular proteins usually pre-exist in certain cell types, contributing to a phenotype that is non-permissive to viral infection. Most of them are induced by interferon (IFN) acting as innate sensors that trigger innate response against a large variety of viruses. Many RF suppress viral replication by directly targeting conserved essential steps of the viral cycle, including viral entry, uncoating, DNA integration, proviral genome transcription, and budding, thus exerting broad antiviral activity. In contrast, some RF inhibit viral pathogens more indirectly by affecting the stability, localization or activity of cellular factors or limiting the availability of cellular resources such as nucleotides needed in the viral replication cycle [51,52]. While each RF uses a distinct mechanism of inhibition, the virus has equally evolved complex strategies to neutralize their inhibitory effect. The majority of these factors were discovered in primates through studies on HIV-1. Apolipoprotein B mRNA-editing enzyme-catalytic polypeptide-like 3G (APOBEC3G), tetherin/BST2, Sterile Alpha Motif and Histidine-Aspartate Domain 1 (SAMHD1), and Tripartite motive 5α (TRIM5α) are some of the best-known HIV-1 RF that have been studied in great detail. [53]. Among them, APOBEC3G (A3G), a cytidine deaminase in which G-to-A hypermutation in the viral genome, inhibiting viral infectivity, was shown to be incorporated into HTLV-1 virions and inhibit HTLV-1 infection without exerting its cytidine deaminase activity. Incorporation of A3G was detected in HTLV-1 virions produced in 293T cells transfected with A3G expression vector, and also by using MT-2, an HTLV-1 producing cell line, which expresses endogenous A3G [54]. Conversely, in another study using HTLV-1 virions produced in 293T expressing exogenous A3G, the authors showed that, despite A3G being efficiently encapsidated in HTLV-1 virions, it was not able to block HTLV-1 infection [55]. By using the same experimental approach, another study demonstrated that HTLV-1 resistance to A3G was partially mediated by a peptide motif in the C terminus of the HTLV-1 nucleocapsid (NC) domain, inhibiting AC3 packaging into nascent virions. [56]. In line with this evidence, DNA sequence analysis of viral genome isolated from HTLV-1 asymptomatic carriers and HAM/TSP patients’ cells has shown that hypermutations occur at very low frequencies, within the range 0.1–5%, suggesting that the packaging of A3G in viral particles is not sufficient to suppress viral infectivity. Interestingly, in HTLV-1-transformed cell lines such as MT-2 and MT-4, and in samples collected from ATL patients and HTLV-1 carriers, sequence analysis of proviral genome indicated that the target sequences of A3G were less frequent in the plus strand of the HBZ coding region than in other coding regions of the HTLV-1 provirus, such as Tax, in part explaining the maintenance of HBZ expression during neoplastic transformation and ATL progression [57]. More recently, a footprint analysis of A3G on the genome of human viruses revealed that A3G editing activity acts both on the antisense and the sense transcripts of the HTLV-1 coding sequence, suggesting that A3G left an evolutionary footprint on the HTLV-1 virus through editing during reverse transcription [58].

Comparatively less information is available for the effect of other RFs on HTLV-1. TRIM5α was originally discovered to be an important determinant of the resistance of monkey cells to HIV-1 infection. Indeed, rhesus monkey TRIM5α (rhTRIM5α), but not human TRIM5α, potently limits HIV-1 infection in Old World monkeys by targeting the viral capsid, thus preventing the uncoating of the viral pre-integration complex. Unlike other RFs, the activity of TRIM5α is not antagonized by an accessory viral protein, since HIV-1 had evolved its capsid to avoid recognition by human TRIM5α, although it is still susceptible to the rhesus monkey version. Rhesus TRIM5α restricts a broad range of retroviruses including HIV-1, HIV-2, N-tropic murine leukemia virus (N-MLV), and equine infectious anemia virus (EIAV).

Interestingly, genome sequence analysis of peripheral blood mononuclear cells (PBMC) from both HAM/TSP patients and AC identified specific TRIM5α polymorphisms associated with proviral load (PLV), indicating a possible role of TRIM5α in HTLV-1 replication [59]. The same correlation was found for TRIM22, another member of the TRIM family [60].

In relation to SAMHD1, recent data obtained from 22 HAM/TSP patients and 61 AC, again demonstrated that the rs6029941 (A/G) polymorphism in host dNTPase SAMHD1 is associated with increased HTLV-1 PLV in HTLV-1-infected individuals, suggesting that the polymorphism could be a factor contributing to the development of the symptoms of the disease [61]. By using HTLV-1 virus isolated from MT-2 supernatants, SAMHD1 has been described to exert its antiviral activity by inducing apoptosis of HTLV-1-infected monocytes, which represents another target of HTLV-1 infection [62].

Besides the classical RF, cellular miR-28-3p was found to suppress viral replication and gene expression in transiently transfected cells with an HTLV-1 molecular clone, by targeting a sequence localized within the viral gag/pol genomic viral mRNA. Indeed, cells expressing a high level of miR-28-3p were found to be resistant to HTLV-1 infection, suggesting a possible antiviral function of this cellular-derived miRNA. Consistent with this hypothesis, a single nucleotide polymorphism within the miR-28-3p target site in the Japanese ATK-1 viral genome strain renders this viral strain relatively resistant to the presence of miR-28, highlighting the role of miRNA in viral transmission [63].

Another cellular factor exerting a potent antiviral activity against HTLV-1 is the MHC class II transactivator, also designated as CIITA, the master regulator of MHC-II genes transcription. First described in our laboratory, CIITA potently suppressed HTLV-1 replication by targeting the viral transactivator Tax-1 [64,65]. Interestingly, the inhibitory activity of CIITA was demonstrated by using HTLV-1 virions produced both in cells ectopically expressing CIITA and, more importantly, in isogenic promonocytic U937 cells that expressed physiological level of CIITA and previously characterized for their efficient or inefficient capacity to support productive HIV-1 infection [66]. Moreover, CIITA as a direct inhibitor of Tax-1 function, was the first RF described to inhibit both viral replication and Tax-1-driven neoplastic transformation. Indeed, CIITA was found to block the persistent activation of NF-κB pathway by Tax-1 not only in cells ectopically expressing CIITA, but more importantly in cells expressing endogenous CIITA [67].

### 2.4. NF-κB Pathway

**Figure 3:**
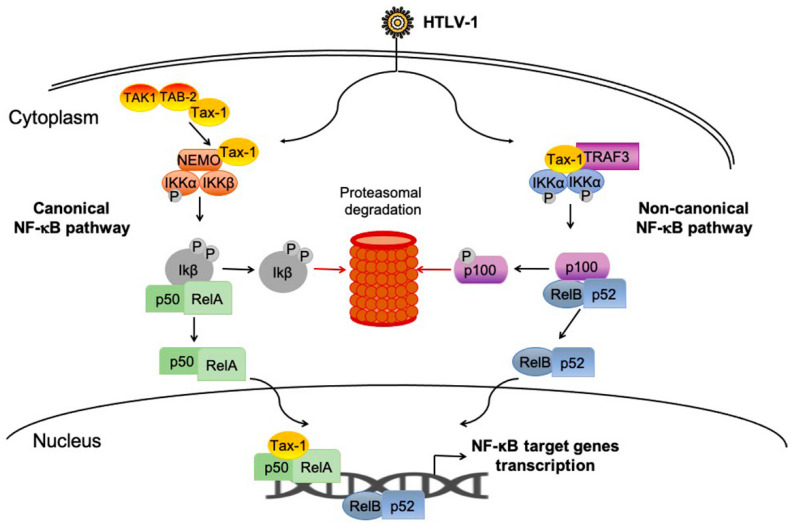
HTLV-1 Tax-mediated NF-κB activation.

The HTLV-1-transformed cell lines (e.g., MT-4, C8166, HUT-102, and M-T2), ATL-derived cells (e.g., F6T, K3T, S1T, and Su9T01) or transiently transfected cells (e.g., Jurkat, HEK293, HeLa cells) are the common cell models used to investigate the role of Tax and HBZ in cell signaling deregulation [11,28]. NF-κB is one of the most extensively studied pathways deregulated by Tax and HBZ, and the Tax activation of the NF-κB pathway is well established as a critical step in the onset of T-cell transformation and development of ATL [68,69,70] (Figure 3). On the other hand, HBZ is required for viral latency and antagonizes many of the activities mediated by Tax, including NF-κB activation [71,72,73]. In transduced 293T and Jurkat cells, HBZ inhibits the expression of cyclin D, a regulator of the G1/S phase transition, interacting with the NF-κB p65 factor [74].

Comparative studies with the less pathogenic HTLV-2 homolog, which expresses a Tax-2 and antisense protein APH-2, have highlighted the exclusive properties of Tax-1 and HBZ that may account for the divergences in HTLV types linked pathobiology [75,76,77]. Tax-1, but not Tax-2, activates both canonical and non-canonical NF-κB pathway; although they share a high amino acidic identity, only Tax-1 presents two leucine-zipper-like regions (LZR), which are required for NF-κB activation and a PDZ-binding domain (PBM) at the C-terminal; they also differ in the cytoplasmic and nuclear domains, which confer a prevalent nuclear distribution to Tax-1 and a prevalent cytoplasmic distribution to Tax-2 [77,78,79]. Tax-1 and Tax-2 also differ in their interactome repertoire and the effects deriving from the interactions [80].

HBZ presents an N-terminal transactivation domain that lacks in APH-2 and inhibits Tax-1 activity more efficiently than APH-2 [81,82,83]. They also differ in the mechanisms that control their intracellular stability, which is regulated by an E3 ubiquitin ligase (UBR5) in HBZ, and by sumoylation mediated by PML nuclear bodies in APH-2 [84,85]. Several genes that are targets of NF-κB, as well as long noncoding RNAs, are differentially expressed in Jurkat Tet-On human T cells expressing subgroups of Tax or HBZ proteins [86].

In vitro cell models have demonstrated the direct interaction of Tax-1 with several factors of the NF-κB pathway, including NEMO/IKKγ, IKKα, TAB2, TRAF6, and the cross-talk factors IKKε and TBK1 [87,88,89]. In cell models, using Tax mutants, it has been possible to characterize the functional requirement of Tax post-transcriptional modifications such as ubiquitination, sumoylation, and phosphorylation in NF-κB activation and binding to the IKK signalosome [90,91,92]. We recently demonstrated using a CRISPR/Cas9 knockout cell model that tumor necrosis factor receptor associated factor 3, TRAF3, a negative regulator of the non-canonical NF-κB pathway, is required for Tax-1-mediated NF-κB activation [70]. Additional cellular factors are required for the efficient Tax NF-κB activation, including optineurin (OPTN) and Tax1-Binding Protein 1 (TAX1BP1), which have been demonstrated to participate in the K63-polyubiquitination of Tax-1. OPTN was shown to interact with Tax in Golgi-associated structures and to enhance its activities in a TAX1BP1-dependent manner [93,94,95]. Recently it has been demonstrated in cell models that SQSTM-1/p62 potentiates Tax activity by facilitating the association of ubiquitin chains with the Tax/IKK signalosome, and the interaction of E3/E4 ubiquitin conjugation factor UBE4B supports HTLV-1 Tax polyubiquitination, NF-κB activation, and cell survival [28,96]. In HTLV-1-infected T cells, Tax activates the early phase of NF-κB activation through interaction with autophagy-regulatory proteins such as Beclin 1, which promotes the recruitment of the IKK complex to an autophagy molecular complex and induces efficient autophagosome formation [97]. Tax protein expression is also stabilized by the NF-κB activity in a positive feedback loop between Tax and NF-κB that requires polyubiquitinilation [98]. 

Tax expression is silenced in the majority of ATL due to genetic alterations in the tax gene or DNA hypermethylation of the 5’-LTR [99]. Nevertheless, NF-κB remains persistently activated in HTLV-1-induced ATL as a consequence of somatic mutations in genes involved in T-/B-cell receptor (T/BCR)-NF-κB signaling and additional epigenetic modification [100]. Recently, the role of mucosa-associated lymphoid tissue lymphoma translocation protein 1 (MALT-1) proto-oncogene has been investigated in ATL cells. MALT-1 is known to participate in the activation of NF-κB by cleavage of inhibitors of NF-κB pathway such as A2 and RelB. In MT-1 and TL-Om1 T cell lines established from ATL patients, which do not express Tax-1, MALT-1 expression is upregulated. Interestingly, the inhibition of MALT-1 expression leads to a reduction in growth and viability of the ATL-derived T cell lines, suggesting MALT-1 as a possible target for future therapeutic approaches in ATL patients [101].

Finally, Tax-mediated NF-κB activation may have an impact on alternative splicing events, enhancing physical and functional interaction between p65 and the DDX17 splicing factor. By constitutive activation of NF-κB pathway, Tax may promote DDX17-dependent splicing regulation enhancing DDX17/p65 recruitment in intragenic region, thus altering splicing target specificity [102].

## 3. Animal Models

Animal models, including mice, rats, rabbits, squirrel monkeys, baboons, macaques, and even fruit flies, although not the natural hosts of HTLV infection, may help in elucidating some aspects of HTLV infection, persistence, host immune response, and diseases-associated developments [103,104,105,106,107]. Examples of the contributions in HTLV studies derived by different animal models are listed in Table 2. In the following sections, we will discuss the most recent advances in the knowledge of HTLV infection and pathogenesis derived by studies in animal models.

### 3.1. Mouse Models

Although immunocompetent murine cells are not productively infected with HTLV-1, xenograft and transgenic mice are widely used for the study of HTLV-1 infection and related diseases. Starting from the late 1990s, when the C3H/HeJ and BALB/c strains were used to establish persistent infection injecting HTLV-1-producing MT-2 cell intraperitoneally in neonatal mice, several HTLV immunocompromised mouse models have been further developed [159,160,161,162]. The development of SCID mice, unable to perform VDJ recombination of B- and T-cell receptors because of a nonsense mutation in the PRKDC (Protein Kinase, DNA-Activated, Catalytic Subunit protein kinase) gene, has generated animals with severe combined immunodeficiency (SCID). These mice allow the engraftment of human cells. By introducing additional genetic mutations, several other types of SCID immunocompromised mouse strains become available, i.e., NOD-SCID mice, in which SCID mutation is present in a non-obese diabetic (NOD) genetic background mouse that shows NK cell dysfunction, low cytokine production, and T- and B-cell deregulation; NSG and NOG mice, in which different mutations in the interleukin-2 receptor common subunit γ (IL2R-γC) leading to a complete loss of T, B, and NK cells, are introduced into the NOD/SCID background; and BALB/c mice deficient in IL2R-γC and the recombinase-activating gene 2 (Rag2) (BRG), which are impaired in T- and B-cell differentiation and have high levels of NK-cell activity [163]. 

SCID xenograft mouse models can reproduce some features of HTLV-1 disease, such as multiple organ engraftments with ATL cells, expression of parathyroid hormone-related protein (PTHrP), a mediator of hypercalcemia in ATL patients, and increased levels of IL2 Rα and β-2 microglobulin [164,165,166]. These xenograft mouse models have contributed to the recapitulation of splenomegaly and lymphoma similar to ATL pathologic features [108]. Several studies have reported the successful engraftments in NOG mice of HTLV-1-transformed cell lines, ATL cells, and PBMCs from asymptomatic HTLV-1 carriers [167,168,169]. Engrafted SCID mice have also been used to assess the tumorigenic potential of ATL cell lines [170]. In NOG mice, a highly tumorigenic ATL cell was selected by serial xenotransplantation of patient leukemic cells and used to study features of ATL such as the involvement of carbonic anhydrase IX (CA9), a membrane-associated enzyme that regulates cellular pH. It was found that CA9 is upregulated and promotes tumorigenicity of ATL-derived cells [171]. A highly penetrant in vivo model of HTLV-1-induced T-cell lymphoma was established by intraperitoneally engrafting immune-compromised NOD/SCID mice with tumorigenic HTLV-1-transformed SLB1 and MET-1 lymphoma T cell lines. In this model, a cooperative role was found between the the viral p30II latency regulatory factor and the cellular TP53-induced glycolysis and apoptosis regulator (TIGAR) in cancer progression, highlighting TIGAR involvement in tumor lymphocyte infiltration [113]. NOD/SCID mice injected with leukemic cells (MET-1) from a patient with ATL were proposed as preclinical in vivo murine models of ATL [172]. In this model, the ATL therapeutic efficacy of selected compounds has been reported. Among other treatments, the efficacy of daclizumab, a monoclonal antibody against the IL-2R-α (CD25), combined with depsipeptide, a member of the cyclic peptide class of HDAC inhibitors, was tested by analyzing the survival of the leukemia-bearing mice and the levels of soluble IL-2R-α and β2μ levels. Both depsipeptide and daclizumab led to inhibition of tumor growth and prolonged the survival of mice with leukemia suggesting its potential use in the treatment of ATL patients [166].

NOD/ SCID mice have recently been used to evaluate a new therapeutic agent for ATL. In this study, NOD/SCID mice were injected with S1T cells, an HTLV-1-infected CD4 + T cell line derived from an ATL patient, and treated with dorsomorphin, an inhibitor of the bone morphogenetic protein (BMP) and AMP-activated protein kinase (AMPK) pathway. The administration of dorsomorphin to NOD/SCID mice proved to be efficient in reducing tumor growth [109]. In another mouse model, the efficacy of monoclonal antibodies in ATL therapy was investigated targeting the matricellular molecule OPN, which is known to participate in cancer processes by interaction with integrins. NSG mice inoculated with ATL cells present increased plasma levels of OPN, and when treated with a monoclonal antibody against OPN tumor growth, invasion and metastasis were inhibited [110]. More recently, the same group examined the antitumor effects of 2′-deoxy-2′-methylidenecytidine (DMDC) and its derivative 2′-deoxy-2′-methylidene-5-fluorocytidine FDMDC in NOG mice inoculated subcutaneously with an ATL-derived cell line. They observed that NOG mice bearing ATL tumor treated with the two compounds resulted in significant inhibition of tumor growth suggesting that nucleosides may be proposed as therapeutic agents in ATL [111].

Antitumor effects of autologous Tax-specific cytotoxic T cell (CTS) have also been tested in NOG mice bearing human primary ATL cells. Tax-CTL treatment led to Tax-specific CTL infiltration in the tumor site, recognition and blocking of the proliferation of autologous ATL cells and prolongation of mouse survival [112], although the reproducibility of this finding is not constant [173].

#### 3.1.1. Humanized Mouse Models

Humanized mouse models derived from mouse xenotransplanted with human cells or engineered to express human genes may be used to study human-specific function in physiological and pathological conditions, most of them related to the human immune system [115]. In HTLV, humanized mouse models are mostly applied for studying the tropism and proliferation of HTLV-infected T cells, but also to elucidate the mechanism of in vivo development of ATL. Humanized mice infected with HTLV-1 may develop ATL, but they are not always consistent in reproducing the human immune responses against HTLV-1 [108,174]. An interesting model was generated by transplanting CD133+ human stem cells into the bone marrow cavity of NOD/Shi-scid/IL-2Rγc null (NOG) mice. These mice, named IBMI-huNOG mice, recapitulate distinct ATL-like symptoms, such as hyperproliferation of CD3+ T cells, clonal proliferation of CD25+ /CD4+ T cells, formation of flower cells in the peripheral blood, hepatosplenomegaly, inflammatory hypercytokinemia, and an adaptive immune response against HTLV-1 [117]. Humanized mice have also been employed to study HAM/TSP neuropathogenesis in an in vivo model. Balb/c-Rag1-hu ^−/−^ γc ^−/−^ (Rag1) and Bone Marrow Liver Thymic (BLT) humanized mice (hu-mice) were engrafted with human CD34+ hematopoietic stem cells and were able to reconstitute human macrophages, dendritic cells, T cells, and B cells [118]. Both models may be susceptible to HTLV-1 infection presenting Tax expression in the spleen and CNS. They also show myelin disruption resembling HTLV-1-associated neuropathogenesis.

Recently, humanized mice that cannot mount an adaptive immune response were obtained by injecting human umbilical-cord stem cells into the livers of immunodeficient NSG mice, and these were applied in the study of T cell tropism and lymphoproliferation of HTLV [175]. In these models, a different tropism of HTLV-1 compared to HTLV-2 was confirmed. HTLV-1 infection is associated with the preferential proliferation of CD4+ T cells, whereas CD8+ T proliferation is associated with HTLV-2. Notably, both viruses are lymphomagenic in mice, in contrast to human leukemia–lymphoma induction, which is typically associated only with HTLV-1 infection, suggesting that the adaptive immune response is critical in conditioning the lymphoma development. A relevant limit in applying the humanized mouse models is the development of graft-versus-host diseases (GVHD), which may cause the early death of mice or inefficiency in recapitulating, within the short lifetime of the mice, the complexity of events that occur in humans over decades of persistent virus prior to ATL development. This limitation was highlighted in a recent study using two humanized mouse models [116]. The authors investigated the role of p8 and p12 regulatory proteins in HTLV-1 infectivity and pathogenicity. p8 and p12, expressed by the open reading frames of the viral genome (orf-I), are required for persistent infection of primary human peripheral blood mononuclear cells in vitro and macaques in vivo [145,146], but are not required in rabbit models of HTLV-1 infection [141,176]. Using NSG-1d mice originated by NOD/SCID/γc ^−/−^ c-kit^+^ engrafted with human tissues and NSG mice implanted with human fetal liver, thymus tissue and stem cells (BLT mice), the authors demonstrated that these humanized mice were highly susceptible to HTLV-1 infection with the rapid polyclonal proliferation of CD4+ CD25+ T cells, similarly to the events in the healthy carrier stage of HTLV infection, although they did not reproduce the monoclonal origin of ATL, as happens in humans [116]. As proposed by the authors, these models may be valid for studying the early phase of HTLV-1 infection and proving interventions that may reduce the CD4+ proliferation induced by the virus. 

In addition to the numerous studies aimed at dissecting the molecular function of the Tax and HBZ viral protein in in vitro cellular model, as summarized in Section 2, interesting contributions towards interpreting their role in vivo in the lymphoproliferative process have also been derived using humanized mouse models. Recently, the contribution of the Tax PDZ binding motif (PBM) to T-cell proliferation was analyzed in humanized mice carrying a human hemato-lymphoid system. It was shown that Tax-PBM enhanced HTLV-1-mediated T-cell proliferation compared to a PBM-deleted mutant, and that this domain is required for T-cell proliferation. Furthermore, comparative transcriptome analyses of T cells derived by humanized mice infected with wt and mutant Tax showed that the absence of PBM is associated with the deregulation of genes involved in T-cell signaling and proliferation, apoptosis induction, and cytoskeletal organization [119]. Taking advantage of humanized mice, the role of HBZ in altering the expression of the receptor activator of NF-κB ligand (RANKL), a regulator of osteoclast differentiation, was evaluated in vivo. In this HTLV-1-infected humanized mouse model, treatment with denosumab, a monoclonal antibody against human RANKL, resulted in reduced bone loss [177].

#### 3.1.2. Transgenic Mouse Models

Transgenic mice have been generated mostly to analyze the oncogenic potential of Tax and HBZ viral protein. Indeed, Tax expression in transgenic mice is sufficient to induce tumors, confirming the in vivo oncogenic potential of Tax [104,120]. An interesting transgenic mouse model was developed by introducing the firefly luciferase gene driven by the HTLV-1 LTR (LTR-LUC) in transgenic Tax mice. The double transgenic Tax-Luc mice develop lymphoma, splenomegaly, hypercalcemia, osteolytic bone selections, and persistent activation of neutrophils [178]. The same team demonstrated that IL-15-deficient Tax-LUC mice developed an aggressive lymphoma and an increased expression of IL-α, thus suggesting IL- 15 and anti IL-1α as potential targets for ATL therapies [121].

To restrict Tax expression to the thymus, Tax transgenic mice have been generated using lymphocyte-specific protein–tyrosine kinase (lck) promoters. These Lck-Tax mouse models develop lymphoma and leukemia after a long latency period of almost 18 months and present most of the characteristics of acute ATL patients [122,123]. Tax transgenic models have also been used to test in vivo the efficacy of ATL therapy [179]. SCID mice injected with spleen cells from Tax transgenic mice developed ATL-like tumors. Treatment of these mice with arsenic/IFN-α or synthetic retinoid ST11926 compound resulted in a significant increase in animal survival [124,180]. In addition, normal syngenic mice injected with ATL cells from Tax-transgenic mice showed inefficient Tax-specific T-cell induction and ATL cells elimination [181].

As for Tax, the in vivo role of HBZ has been studied in HBZ transgenic (HBZ-Tg) mice. HBZ is the only regulatory/accessory gene encoded by HTLV-1 to be expressed in all ATL patients and necessary for the proliferation of ATL cells [23]. Mice expressing HBZ under the Granzyme B promoter (Gzmb-HBZ) developed lymphoproliferative disease and hypercalcemia [125]. HBZ transgenic models in which HBZ expression is restricted to CD4+ are preferentially used to study the inflammatory process correlated with HTLV-1–mediated pathogenesis. These HBZ-Tg mice develop systemic inflammation and T-cell lymphoma [182], and show higher levels of the immunosuppressive cytokine IL-10 and dysfunctional Treg cells [23,126]. In an interesting HBZ-Tg-based model, it was recently demonstrated that HBZ plays a pivotal role in dysregulating the cytokine signaling modulating the IL-10/JAK/STAT signaling pathway. As expected, in HBZ-Tg the loss of IL-6 and expression of IL 10 accelerates inflammation and lymphomagenesis [25]. HBZ-Tg-derived T-cell lymphoma has also been used to establish an HBZ-induced T cell line, named Ht48, which has been used to test an HBZ-targeted HTLV-1 vaccine. This model identified a candidate peptide (HBZ157-176) for vaccine development by using rVV-vaccinated mice [127].

PBMC-humanized NSG mice and HBZ-transgenic (Tg) mice, which develop systemic inflammation, were recently used to validate the efficacy of administration of pentosan polysulfate (PPS), a semisynthetic glycosaminoglycan, to counteract HTLV-1 infection and pathological sequelae. PPS blocked HTLV-1 infection in huPBMC NSG and suppressed the development of dermatitis and lung damage in HBZ-Tg mice, supporting the therapeutic use of PPS in the treatment of HTLV-1-induced inflammatory diseases [183]. 

Tax-transgenic (Tax-Tg) and HBZ Tg mouse models have contributed to identifying functional ATL stem cells (ATLSC) and determining that c-kit, a common surface marker of ATLSCs, is a key regulator of ATL disease initiation and progression [128]. Unexpected results were obtained using a double transgenic mouse model expressing both Tax and HBZ in CD4+ cells. These mice developed T-cell lymphoma but not ATL-like leukemia, suggesting that the balancing effect of Tax/HBZ expression is critical for oncogenic outcome [129]. Mouse models of acute-type ATL can be rapidly generated by transplanting in vitro-induced T cells that have been retrovirally transduced with HBZ. In this model, it is possible to study the cooperative action of HBZ and host factors in contributing to ATL development [184].

### 3.2. Rat Models

Rat models have been useful in the study of HAM/TPS pathology. HTLV-infected Wistar-King-Apekman (WKA) rat strain develops spastic paraparesis and clinical symptoms similar to the humans with HAM/TPS [130]. Rats have also been used to study mother-to-child transmission (MTCT) of HTLV-1 Recently an MTCT model was developed using orally HTLV-1-infected rats that did not have antibody responses against viral antigens. In this model, rats inoculated with ILT-M1, an IL-2-dependent HTLV-1-infected T cell line derived from an HAM/TSP patient, transmitted HTLV-1 to their offspring at a high rate (50–100%), and the rate of transmission correlated with the PVL of the infected mother rats [131]. This model has been also proposed for studying the neutralizing potential of antibodies against HTLV-1 envelope gp-46 (LAT27) through antibody-dependent cellular cytotoxicity in MTCT [132,185].

The role of host factors in supporting viral infection has also been investigated in rat models. Human CRM1 (hCRM1) protein, a member of the importin β family, acts as a cofactor of Rex-dependent viral mRNA transport. Transgenic CRMI1 rats intraperitoneally inoculated with HTLV-1-infected cells exhibited a much higher HTLV-1 viral production than wild type rats, and presented more extensive invasion of the thymus by HTLV-1, supporting the in vitro evidence of the key role of CRM1 in HTLV-1 infection [133]. Rat models were also used to test the effect of vaccines based on HTLV-1 Tax-specific cytotoxic T lymphocyte immunity response, the oncolytic potential of vaccinia viruses (VVs) and the ability of siRNA Tax downregulated HTLV-1-infected cells to develop tumors in T-cell-deficient nude rats [134,135,136]. These studies confirm the significant roles of Tax in activating cytotoxic host immune response to the virus and in the survival of infected cells in vivo.

### 3.3. Rabbit Models

Rabbits are well established and reproducible models to study HTLV-1 transmission, immune responses, and viral determinants required for HTLV infection. Rabbits can be infected with HTLV, but do not develop HTLV-associated diseases; nevertheless, they produce a persistent infection and represent a useful animal model for studying the early steps of infection [106,149,186]. New Zealand White (NZW) rabbits injected with an HTLV-1 carrying a PBM-deleted form of Tax-1 showed that this domain was important for the establishment and maintenance of persistent infection [137]. A similar model was used to demonstrate in vivo that HBZ enhances infectivity and persistence and that the HBZ leucine zipper domain is critical for HBZ functional activity, whereas HBZ is dispensable for immortalization/transformation of primary T lymphocytes in cell culture [138]. In rabbits, APH-2 studies have demonstrated that compared to HBZ, APH is not required for viral persistence [140]. In addition, rabbit models have been successfully applied to demonstrate that the HTLV-1 accessory proteins p12, p13 and p30 are necessary to establish the infection and maintain viral loads in vivo [141,142,143].

Recently the NZW rabbit model was also used to study epigenetic regulation of HTLV-1 gene expression in vivo, demonstrating that the CCCTC binding site present in the overlapping p12 and HBZ sequences of the HTLV-1 genome is dispensable for persistent infection [75]. Particularly worthy of note are studies in rabbit models that have contributed to better defining the differences in the HTLV-1 and HTLV-2 tropisms. It has been possible to determine that at early steps of infection, at the entry step, the tropism is almost the same, represented by CD4+ and CD8+ T cells, although, consistent with reports in humans, HTLV-1 establishes a more robust infection in both CD4+ and CD8+ T cells, compared to HTLV-2 [139].

### 3.4. Non-Human Primate Models

Non-human primates are susceptible to HTLV-1 infection and develop HTLV-1-associated diseases, including leukemia. Squirrel monkeys, cynomolgus monkeys, rhesus macaques, and pig-tailed macaques have been used to study HTLV immune response, viral persistence, and ATL-like disease. In squirrel monkey Saimiri sciureus injected with HTLV-1-immortalized PBMCs, the spleen and lymph nodes were shown to serve as major reservoirs for HTLV-1 [187]. p12, p30, and HBZ have been found to be essential for establishing and maintaining HTLV-1 infection in macaques, but not in rabbits [145]. Furthermore, by inoculating macaques intravenously with lethally γ-irradiated B-cell lines producing mutated viral clones, it was shown that p12 and p8 are necessary for efficient viral persistence and spread [146]. The non-human primate models have contributed to recapitulating the initial steps of viral infections, including viral genome reverse transcription and persistent clonal expansion of infected cells [147]. Squirrel monkeys, as well as macaque rhesus, have also been used to evaluate the immunogenicity of experimental vaccines against HTLV-1 [127,144]. However, due to the high cost and restrictive regulations related to their application in experimental research, non-human primate models remain of very limited use in the study of HTLV-1.

### 3.5. Transgenic Fly Model

A Tax and HBZ transgenic Drosophila melanogaster fly model was recently proposed as a suitable model for studying HTLV-I transformation, persistence, and epigenetic modification. The in vivo fly model demonstrated that Tax activates the chromatin polycomb repressive complex 2 (PRC2), which acts on the regulation of the expression of genes involved in cell survival, proliferation, or apoptosis. In this model, HBZ does not induce transformation or NF-κB activation, but its expression abolishes Tax-mediated PRC2 activation in flies expressing both Tax and HBZ [107,188].

## 4. HTLV-1-Related Virus and Animal Models of Leukemogenesis

### 4.1. HTLV-1/BLV Models

Bovine leukemia virus (BLV) is a retrovirus closely related to HTLV-1 that causes B-cell lymphoma in ~5% of infected animals and has been proposed as a model for investigating the transmission, latency and pathogenesis of both BLV and HTLV [149,152]. In addition to cattle, BLV may infect sheep, and both species can develop leukemia and lymphoma. Sheep experimentally infected with BLV represent an interesting model for studying leukemia/lymphoma, as they systematically develop leukemia/lymphoma in a shorter period of ∼20 months. In this model, it is possible to monitor all stages of the viral-induced disease, from infection, through asymptomatic stages, to terminal leukemia, recapitulating the development of HTLV-1-associated human malignancy. BLV sheep models have contributed to defining the viral and host determinants for viral persistence and latency and to exploring the efficacy of potential cancer treatment and viral vaccine [150,151,189]. Recently, comparative analyses of HTLV-1/BLV proviral integration sites in the host genomes were performed from the primary tumors and asymptomatic stages of the infection using high-throughput sequencing mapping and RNAseq [148]. This study demonstrated that HTLV/BLV proviruses are integrated close to cancer driver genes, the expression of which may be cis-perturbed, contributing to malignant progression in the polyclonal expansion of the infected cells. Proteome analysis of sheep lymphocytes in the course of BLV-induced leukemia identified novel potential protein markers of disease progression such as spleen trypsin inhibitor, CXCL4/PF-4, thrombospondin, vasodilator-stimulated phosphoprotein, and the fibrinogen alpha chain that are worthy of further investigation in HTLV-induced leukemia [190]. Defining the genetic and epigenetics factors that characterize the sheep BLV leukemia also offers the opportunity to test antiviral gene target therapies.

### 4.2. STLV Models

STLV-1 naturally infects non-human primates such as the Japanese rhesus macaque, Mandrillus sphinx, and Papio anubis and, like HTLV-1, causes ATL adult T-cell leukemia and lymphoma. [153]. Compared to HTLV-1 infection, Japanese monkeys infected by STLV-1 present similar host immune responses to viral protein and similar clonality of virus-infected T cells, representing a valid model for studying persistent infection and for developing immune-based therapy and prophylaxis [153]. Administration of anti-CCR4 antibodies to STLV-1-infected Japanese macaques resulted in a reduced proviral load in vivo, which is consistent with its efficacy in patient ATL treatment [154]. Furthermore, a long-lasting decrease in the number of STLV-1-infected cells in vivo was observed when Japanese macaques were treated with the humanized anti-CCR4 monoclonal antibody mogamulizumab, which enhances T-cell responses to viral antigens and suppresses CCR4+ Treg cells [155]. Recently, the effect of monoclonal antibodies on CD8 and CD16 was also explored in Japanese macaques infected with STLV-1; although not conclusive, the results suggested that depletion of CD8+ cells was able to modify the clonal proliferation of the infected cells [156].

In Papio papio baboons naturally infected with STLV-1, it was observed that the combined treatment with valproate, an inhibitor of histone deacetylases, and azidothymidine, an inhibitor of reverse transcriptase, caused a strong decrease in the proviral load and an increase in the STLV-1 specific cytotoxic T-cell population [157]. Due to the similarity with the human immune system, STLV-1-infected baboons have been proposed as a model for testing HTLV-1 vaccines based on immunogenic Tax epitopes. In this model, distinct Tax epitope-rich regions have been shown to be targeted by STLV-1-specific CD8+ T cells [158].

## 5. Conclusions

Despite recent advances in ATL treatment, including multiagent chemotherapy, allogeneic hematopoietic stem cell transplantation, anti-CCR4 monoclonal antibody, and antiviral therapy, the ATL prognosis remains poor. Cell and animal models, although they suffere limitations with respect to replicating HTLV-1 human infection and related diseases, have been and still remain extremely useful models for identifying new key host and viral factors required for HTVL replication ad pathogenesis. It is expected that these models will be improved following the recent advancement in cell-based technologies. Genome editing by CRISPR/Cas9 system targeting HTLV integrated genomes, single-cell analyses, immunogenic peptide design, RNA-based therapy, and improvement in drug delivery are all expected to contribute to the future development of novel and more effective therapies for HTLV-1 related diseases.

## Figures and Tables

**Table 1:** Commonly used cell lines for studying HTLV-1.

Origin	Name	IL-2- Dependency Growth	Phenotype	References
ATL derived cells	ATL-2	independent	CD4+ CD3-	[26]
	ATL-T	independent	CD4+	[26]
	ATL-35T	independent	CD4+	[27]
	ATL-55T	dependent	CD4+	[27]
	ED	independent	CD4+	[27]
	F6T	independent	CD4+ CD25+	[28]
	K3T	independent	CD4+ CD25+	[28]
	MT-1	independent	CD4+ Tax-	[11]
	TL-Om1	independent	CD4+ Tax-	[28]
	S1T	independent	CD4+ CD25+	[28]
	Su9T01	independent	CD4+	[28]
Chronically infected	C91-PL	independent	CD4+	[26]
	MS-9	dependent	CD4+	[29]
HTLV-1-transformed cell lines	MT-2	independent	CD4+ CD25+ FoxP3+	[26,29]
	MT-4	independent	CD4+	[27]
	HUT-102	independent	CD4+ Tax+	[30]
	C8166	independent	CD4+	[31]
	SP	dependent	CD4+ CD8+ CD3+	[26]
Transiently transfected cells	Jurkat	independent	CD4+ CD3+	[31]
	HEK293	independent	CD4-	[31]
	HeLa	independent	CD4-	[11]

**Table 2:** Exemplification of animal models contribution for HTLV studies.

Animal Models	Contribution	References
SCID Mice	ATL-like pathologic features; viral proliferation; ATL therapeutic drugs; tumorigenic potential of HTLV-infected or ATL cells	[108,109,110,111,112,113,114]
Humanized mice	ATL development, immune response; HAM/TSP neuropathogenesis, HTLV-1 and HTLV-2 cell tropism; Tax functional domains	[115,116,117,118,119]
Transgenic mice	Tax and HBZ role in HTLV-1 pathogenesis; vaccine development; ATL stem cells	[23,25,104,120,121,122,123,124,125,126,127,128,129]
Rat	HAM/TPS disease, MTCT, CTL response	[130,131,132,133,134,135,136]
Rabbit	HTLV persistence and viral requirement, *distinct pathogenesis* of *HTLV*-*1* and *HTLV*-*2*	[75,137,138,139,140,141,142,143]
Monkey	Viral persistence, immune response and vaccination, viral protein requirement for HTLV infection	[144,145,146,147]
BLV/HTLV	Virus Transmission, latency, leukemogenesis genome integration	[148,149,150,151,152]
STLV	Viral clonality, immuno-based therapies	[153,154,155,156,157,158]
